# Zoonotic Problems Reported by Sheep and Goat Farmers and Factors Potentially Contributing to the Occurrence of Brucellosis among Them

**DOI:** 10.3390/ijerph191610372

**Published:** 2022-08-20

**Authors:** Daphne T. Lianou, Efthymia Petinaki, Charalambia K. Michael, Anargyros Skoulakis, Peter J. Cripps, Eleni I. Katsarou, Elias Papadopoulos, Charalambos Billinis, Angeliki I. Katsafadou, Vasia S. Mavrogianni, Mariangela Caroprese, George C. Fthenakis

**Affiliations:** 1 Veterinary Faculty, University of Thessaly, 43100 Karditsa, Greece; 2 Department of Microbiology, University Hospital of Larissa, 41110 Larissa, Greece; 3 Laboratory of Parasitology and Parasitic Diseases, School of Veterinary Medicine, Faculty of Health Sciences, Aristotle University of Thessaloniki, 54124 Thessaloniki, Greece; 4 Faculty of Public and One Health, University of Thessaly, 43100 Karditsa, Greece; 5 Department of Agriculture, Food, Natural Resources and Engineering (DAFNE), University of Foggia, 71122 Foggia, Italy

**Keywords:** biosecurity, brucellosis, goat, health management, sheep, zoonosis

## Abstract

The study aimed to investigate the occurrence of zoonotic problems reported by dairy small ruminant farmers in Greece and to study potential associations with socio-demographic characteristics of the farmers and management practices applied in the farms. A countrywide investigation was performed in 325 sheep and 119 goat farms in the 13 administrative regions of Greece. The selected farms were visited and interviews were conducted with respective farmers. The occurrence of zoonotic problems in the farmers was recorded. A total biosecurity score (0–6) was devised, based on biosecurity practices followed in farms. Sixty-seven farmers (15.10%, 95% confidence intervals (CI): 12.1–18.7%) reported experiencing a zoonotic problem. Most of the farmers (*n* = 57) (85.1%, 95% CI: 74.76–91.7%, of those with a zoonotic problem) (12.8%, 95% CI: 10.0–16.3%, of all) reported that the zoonotic problem had been brucellosis. Odds ratio for the occurrence of brucellosis in goat farmers was 1.879 (95% CI: 1.051–3.359) compared to the occurrence of the infection in sheep farmers (*p* = 0.033). For the outcome ‘occurrence of brucellosis’ in sheep farmers, the application of hand-milking, the availability of a separate lambing area and the presence of cats in the farm emerged as significant (*p* < 0.01); for the same outcome in goat farmers, only the availability of a separate kidding area emerged as significant (*p* = 0.001). The mean biosecurity score in farms in the continental area of the country was significantly higher than in the islands: 3.45 ± 0.05 versus 2.76 ± 0.28, respectively (*p* = 0.006), whilst there was also a significantly higher score in farms, where the farmer reported occurrence of brucellosis: 3.68 ± 0.15 versus 3.34 ± 0.06 in farms, where the farmer did not report such an incident (*p* = 0.042). In farms, where the above predictors prevail, farmers should be warned of an increased potential risk for human infection and biosecurity measures should be implemented and tightened.

## 1. Introduction

Dairy sheep and goat farming is the most important sector of the agricultural industry in Greece, with a significant annual milk production. Recent data of the Hellenic Milk Board have indicated a total of 51,750 farms (38,717 sheep flocks and 13,031 goat herds) that had produced and delivered milk during the calendar year 2019 [1]. There are approximately 8,400,000 sheep and 3,600,000 goats in the country, which account for 6.5% and 22.0% of respective total animal numbers in Europe. Milk produced from these animals amounts to approximately 15% of the total European milk production from small ruminants [2], which confirms Greece as a significant producer of milk of small ruminants in Europe. Small ruminant farming is the stronger branch of animal production industry in Greece, producing about 18% of the total income of the primary sector income [3]. In over 98% of farms, sheep and goat farming in Greece refers to dairy production. In fact, in the country, milk production from sheep and goats exceeds by far the milk production from cattle [4].

A variety of zoonotic agents can be transmitted from small ruminants to farmers [5]. These include bacterial (e.g., *Brucella* spp., *Campylobacter* spp., *Leptospira* spp., *Salmonella* spp., *Coxiella burnetii*), fungal (e.g., *Trichophyton* spp., *Microsporum* spp.), viral (e.g., contagious ecthyma virus), protozoan (e.g., *Cryptosporidium* spp., *Giardia* spp.) or metazoan (*Fasciola hepatica*) pathogens. Pathogen transmission can take place through direct contact with the infected animals, although other modes or transmission, e.g., by the aerogenous route, can also apply. Some of these infections, e.g., leptospirosis or Q fever, which can involve various organs (liver, lungs etc.), would develop with non-specific clinical signs (fever, headache, fatigue, nausea, vomiting etc.), creating a diagnostic problem that requires detection of specific antibodies in paired-samples for accurate diagnosis [6,7]. Some others, e.g., cryprosporidiasis or giardiasis, can be present as asymptomatic infections and most often would be self-limiting in healthy individuals [8].

Among the various infections, brucellosis (*Brucella melitensis*) is likely the most important, due to high incidence of human infections. Annual incidence can reach up to 200 cases per 100,000 people and a figure of 500,000 new cases of the infection globally is considered as an acceptable estimate [9]. Small ruminants are the main reservoir for the pathogen and people can be infected by direct contact with animals or indirectly through consumption of contaminated animal products. In people, brucellosis is a significant occupational infection; professionals who come in contact with animals or animal material, e.g., veterinarians, farmers, slaughterhouse workers, butchers, laboratory personnel, are at high risk for infection [10]. In Greece, the number of cases of brucellosis in people has remained stable at around 100 to 150 annually since 2009 [11,12].

Internationally, there are some studies that have focused on risk factors for development of zoonotic infections by small ruminant farmers. For example, De Lange et al. [13] and Meadows et al. [14] have studied Q fever infections in Dutch and Canadian, respectively, farmers; Sanhueza et al. [6] have worked in New Zealand and focused on *Leptospira* infections; and Yadav and Aggarwal [15] have worked on brucellosis in India. All of these studies were of limited geographic extent within the respective countries. At worldwide level, there is little knowledge about possible associations of management factors applied in small ruminant farms or of characteristics of such farmers with the development of zoonotic diseases.

The present study refers to an extensive countrywide investigation performed in 444 dairy small ruminant farms (325 sheep flocks and 119 goat herds) throughout Greece. The study aimed to investigate the occurrence of zoonotic problems reported by sheep and goat farmers in Greece and to study potential associations with socio-demographic characteristics of the farmers and management practices applied in the farms.

## 2. Materials and Methods

### 2.1. Farm Visits and Interviews

**Figure 1:**
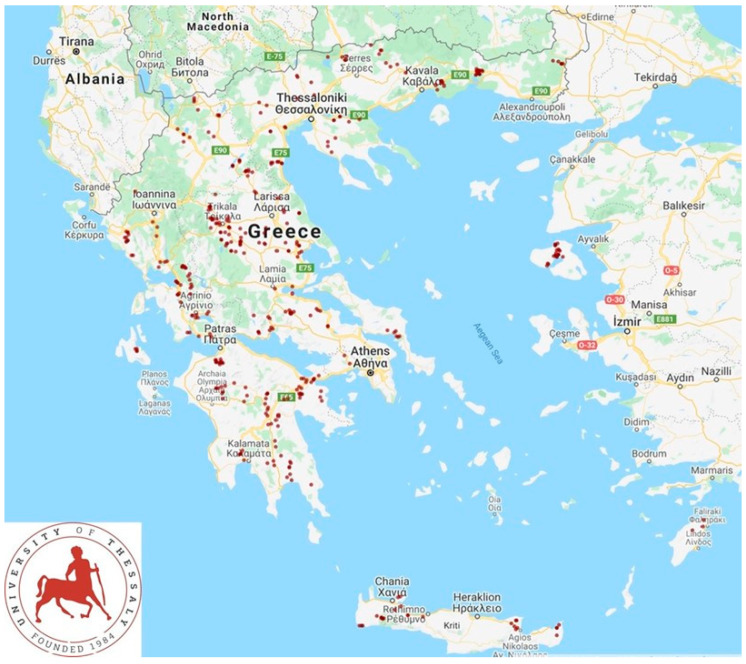
Location of 444 small ruminant farms around Greece, which were visited during a countrywide study for interview of farmers regarding zoonotic problems.

In total, 325 sheep flocks and 119 goat herds in the 13 administrative regions of Greece (Figure 1) were included into the study and visited for collection of information. The field work was carried out from April 2019 to July 2020. Veterinarians active in small ruminant health management around Greece were contacted by telephone and asked if they wished to collaborate in the investigation; in total, 48 veterinarians were contacted and, of these, 47 (97.9%) agreed to collaborate. Farms were selected by the collaborating veterinarians on accessibility basis and the willingness of the farmers to accept a visit by university personnel for an interview. Each of these veterinarians had a stable, although not contractual, association with the respective farm, among those selected for visit, and were responsible for their decisions and actions in relation to the health and welfare of the animals therein, in full accord with the relevant veterinary conduct codes [16,17]. Visits had been scheduled to 446 farms, but on two occasions, whilst the investigators and the accompanying veterinarians had already arrived at these farms, the respective farmers refused to collaborate (both mentioned that they did not have enough time available to receive a visit). The principal investigators (authors D.T.L. and G.C.F.) visited all the selected farms.

During the visit, an interview of the farmer was performed by using a detailed questionnaire [18]. The same person (author D.T.L.) conducted all the interviews. Farmers were asked if a zoonotic problem with confirmed diagnosis, based on clinical signs or results of laboratory examinations, had happened to them; in case of a positive answer, they were asked to name the problem(s).

No relationship had been established between the interviewer and any of the farmers prior to the study. The interviewer was introduced to the farmers by the veterinarian accompanying on each occasion and the senior investigator (author G.C.F.); the farmers were informed about the identity and the employment of the interviewer and that the work was part of her doctoral thesis. The characteristics of the interviewer were also identified. Farmers who asked for clarifications on the questions were given those immediately by the interviewer (full details described by Lianou et al. [18]). The mean value (±standard error of the mean) of the duration of the interview was 63.6 ± 0.3 min. [18]. After completing the interview, no repeat visits were made to the farms.

### 2.2. Data Management and Analysis

The following socio-demographic characteristics of farmers (Table S1) were recorded: gender, length of farming activity, professional involvement in farming, daily time spent at the farm, education and family farming-tradition. With regard to management practices applied in the farm, the following variables (Table S1) were taken into account: animal species farmed, management system applied in the farm, transhumance, application of machine- or hand-milking, number of animals in the farm, breed of animals in the farm, annual number of veterinary visits to the farm, daily number of milking sessions applied in the farm, annual number of disinfections performed, use of reproductive control techniques, availability of a separate lambing/kidding area, vaccination of animals against *B. melitensis* infection and distance of farm from hospital or clinic. The presence of other animals (large ruminants, pigs, dogs, cats or equines) in the farm (Table S1) was also recorded. All the above were recorded during the interview.

The following biosecurity-related factors applied in the selected farms were evaluated for potential association with the reported occurrence of zoonotic problems by the respective farmers: quarantine of new animals arriving at the farm, isolation of sick animals at the farm, means for disposal of carcasses of animals that died in the farm, presence of a ditch at the entrance of the farm, presence of a fence or a wall around the farm and carrying out disinfections in the farm. For each of the above factors, the value of ‘1′ was assigned for each practice aligning with biosecurity rules, whilst the value of ‘0′ was assigned for each practice opposing biosecurity rules (Table S3). The values assigned were summed and a total ‘biosecurity score’, ranging from 0 to 6, was produced for each farm.

Data on farm location were collected in the field using hand-held Global Positioning System units; the geo-references were resolved to specific farm level. ArcGIS software (ESRI; Redlands, CA, USA) was employed for description and analysis of spatial information, specifically the distance of each farm from the nearest hospital or clinic.

Data were entered into Microsoft Excel and analyzed using SPSS v. 21 (IBM Analytics, Armonk, NY, USA).

Biosecurity scores were compared among cohorts of farms by using analysis of variance. The importance of the association of each biosecurity-related factor with occurrence of the diseases was assessed by using cross-tabulation with Pearson’s chi-square test.

The outcome of ‘occurrence of brucellosis’ in farmers was considered. Basic descriptive analysis was performed. Exact binomial confidence intervals (CI) were obtained. For the evaluation of the geographical location of farms, four area clusters were created: central, islands, north and south of the country. For the evaluation of the management system applied in the farms, the classification of the European Food Safety Authority [19] was used: intensive, semi-intensive, semi-extensive and extensive management system. Initially, the findings were described according to animal species farmed (sheep/goats) and the geographical location of the farm. In total, 18 variables (Table S1) were evaluated for potential associations with the above outcome. Initially, the importance of the association was assessed by using cross-tabulation with Pearson’s chi-square test and with simple logistic regression without random effects. Separate analyses were performed for sheep and goat farmers. For this specific analysis, farms located in the islands of the country (bar farms on the island of Lesvos) were not included in these analyses, as in those locations brucellosis has been eradicated; no movements of animals from the continental part of the country into these locations are allowed and no vaccinations against the infection are performed there. Multivariable models were then created. Initially, all variables which achieved a significance of *p* < 0.2 in the univariable analyses were offered to these models (Table S2). Variables were removed from the initial model by backwards elimination. The *p* value of removal of a variable was assessed by the likelihood ratio test, and for those with a *p* value of >0.2 the variable with the largest probability was removed. This process was repeated until no variable could be removed with a *p* value of >0.2. The variables required for the final multivariable model for each species are shown in Table S2.

Finally, the number of small ruminant farmers in the country (in the years 2019–2020) who had had brucellosis at some point during their professional life was estimated based on the reported incidence of the infection in farmers in the among the 444 farms visited in this study. Population incidence was calculated, broken down according to the area of the country (central, islands, north, south) and the animal species farmed (sheep, goats), taking into account the number of farms available in the country in 2019.

In all analyses, statistical significance was defined at *p* < 0.05.

## 3. Results

**Figure 2:**
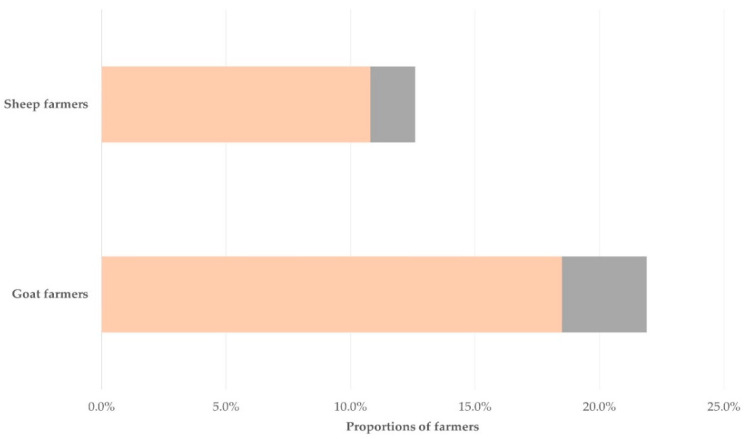
Proportions of sheep and goat farmers who reported occurrence of a zoonotic problem, during a countrywide study in Greece (light brown color: report of occurrence of brucellosis, gray color: report of occurrence of other zoonotic problem).

Sixty-seven farmers (15.1%; 95% confidence intervals (CI): 12.1–18.7%) reported that they had experienced a zoonotic problem (Table 1). Most of the farmers (*n* = 57; 85.1%, 95% CI: 74.7–91.7%, of those with a zoonotic problem—12.8%, 95% CI: 10.0–16.3%, of farmers interviewed) reported that the zoonotic problem had been brucellosis (Table 1); this was similar in sheep (*n* = 35) and goat (*n* = 22) farmers: 85.4% (95% CI: 71.6–93.1%) and 84.6% (95% CI: 66.5–93.9%) of all reports, respectively (*p* = 0.93) (Figure 2). Odds ratio for the occurrence of brucellosis in goat farmers was 1.879 (95% CI: 1.051–3.359) compared to the occurrence in sheep farmers (*p* = 0.033). Moreover, three farmers reported that they had been infested with fleas and one farmer that they had been infested with ticks.

Most cases of brucellosis were reported by farmers in the continental area of the country: 14.5% of farmers versus 0.8% of farmers in the islands (*p* = 0.006). In contrast, no such difference was evident between the three parts of the continental area of the country (*p* = 0.58) (Table 2).

All the details and the full results of the various univariable associations performed for the outcome of ‘occurrence of brucellosis’ are provided in Table S4. Results of separate analyses for sheep and goat farmers are presented.

**Figure 3:**
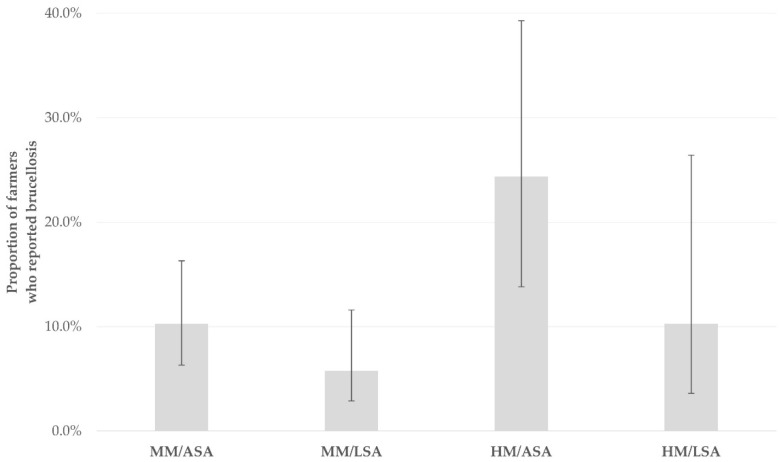
Proportions of sheep farmers who reported occurrence of brucellosis during a countrywide study in Greece, in association with practices employed in the respective farms (MM: machine-milking, HM: hand-milking, ASA: availability of separate lambing area, LSA, lack of separate lambing area).

For the outcome ‘occurrence of brucellosis’ in sheep farmers, the univariable analysis indicated significant association with four variables; these were the increased length of farming activity, the application of hand-milking, the availability of a separate lambing area and the presence of cats in the farm (Table S4). Of these, the three factors that emerged as significant in the multivariable analysis were the application of hand-milking, the availability of a separate lambing area and the presence of cats in the farm (*p* = 0.005, *p* = 0.009, *p* = 0.007, respectively) (Table 3, Figure 3). For the same outcome in goat farmers, the univariable analysis indicated significant association with two factors: transhumance and the availability of a separate kidding area (Table S4). The latter was the only factor that emerged as significant in the multivariable analysis (*p* = 0.001) (Table 3).

The mean (±standard error of the mean) biosecurity score in the selected farms in the study was 3.39 ± 0.06. The mean biosecurity score in farms in the continental area of the country was significantly higher than in the islands: 3.45 ± 0.05 versus 2.76 ± 0.28, respectively (*p* = 0.0006). Biosecurity score 6 was not obtained in any farm in the study, whilst score zero (i.e., for none of the evaluated biosecurity-related factors, practices aligning with biosecurity rules were followed) was obtained in nine farms (2.0%), two of which were in the continental area and seven in the islands (*p* < 0.0001). There was also a significantly higher score in farms where the farmer reported occurrence of brucellosis: 3.68 ± 0.15 versus 3.34 ± 0.06 in farms, where the farmer did not report such an incident (*p* = 0.042). Among the various components of the devised biosecurity score, isolation of sick animals at the farm was more frequently practiced by farmers who reported occurrence of brucellosis: 96.5% among those farmers versus 82.4% among farmers who did not report such an occurrence (*p* = 0.007); for the other components of the biosecurity score, no significant difference was seen between farmers who reported or did not report occurrence of brucellosis (*p* > 0.17).

The total number of small ruminant farmers in Greece who had had brucellosis at some point during their professional life was estimated to be 6471 (min. 3513, max. 12,152) (Table S5). This is approximately 12.5% (95% CI: 6.8–23.5%) of total small ruminant farmers and 0.06% (95% CI: 0.03–0.11%) of the total population in the country.

## 4. Discussion

The present study has investigated the occurrence of zoonotic problems in small ruminant farmers in an extensive countrywide investigation in 444 farms. Farmers from all regions of Greece were included in the study; that way, situations and conditions present in all the parts of the country were taken into account. In order to minimize possible bias, the study also used consistent methodologies and ensured that specific tasks were always performed by the same investigators.

Although this approach had the advantage of the extensive area of investigation throughout the country, there were nevertheless limitations regarding the diagnosis of the zoonotic problems. These included the non-specific clinical signs or the development of the infections in an asymptomatic form, as mentioned previously. Hence, one may postulate that there might have been an underestimation of zoonotic problems diagnosed in these farmers. Indeed, in the studies of Antoniou et al. [20,21], various other zoonotic infections were diagnosed in rural populations, some of which could have been transmitted from small ruminants.

In contrast, brucellosis develops with acute and severe clinical signs and requires medical attention for management of the case [22]; hence, specific diagnosis was achieved and the affected farmers became aware of the problem. This contributed to its higher reporting than other zoonotic problems among this cohort of people. The infection is a well-established zoonotic disease, associated with professionals working with ruminants (e.g., farmers, milkers and veterinarians) [23,24].

In Greece, both control and eradication programs against brucellosis of small ruminants are applied, depending on the geographical area of the country [11,25]. Brucellosis was reported significantly more frequently in the continental area of the country and very infrequently in the islands, in line with previous (over 25 years ago) findings of testing people living in the island of Crete, in which antibodies against *Brucella* spp. were detected in <1% of people sampled [20]. With regard to animal infections, recent findings have indicated that the rate of new infected farms in the vaccination zone was significantly higher than in areas where eradication is carried out: 9 per regional unit (interquartile range: 2–23) versus 0 per regional unit (interquartile range: 0–2), respectively [26]. In 2016, the proportion of small ruminant farms with seropositive animals in three regions of the continental part (vaccination area) of the country was reported to be 4.6% overall (9.9% in Thessaly [27], 6.9% in Western Macedonia [28] and 1.8% in Peloponnese [29]), whilst in Crete no cases of the infection were recorded [11]. These differences are likely the consequence of the long-standing eradication scheme applied by the national Ministry of Agricultural Development and Food of Greece against the infection in small ruminants in the islands of the country based on test-and-slaughter policy for all infected animals; in contrast, in the mainland part of the country a control program based on mass vaccination of females is being implemented (vaccination zone with *B. melitensis*, strain Rev-1, administered by the conjunctival route) [11]. As part of that eradication scheme in the islands, slaughter of all seropositive animals and prohibition of imports of animals from other areas have been imposed.

In a relevant study, Jelastopulu et al. [30] reported that the incidence of brucellosis in local people decreased significantly after successful implementation of a control program in small ruminants: 10.3 cases per 1000 people population in 1997–1998, before the vaccination, versus 0.3 cases per 1000 people population in 2000–2002, after initiation of vaccination [30], findings which align with the countrywide trend in Italy from 1997 to 2016 [31]. The reverse situation has also been reported: after cessation of anti-brucellosis vaccinations of small ruminants in Greece in 1996, the incidence of the infection in humans increased rapidly, with a positive correlation with the increase of the prevalence in sheep and goats [32]. In the continental area of the country, brucellosis is endemic in small ruminants [33], although a vaccination program of the animals is currently applied. This was reflected in the increased number of reports of the infection by farmers, given that in endemic areas, the incidence rate in people can be as high as 10% [34].

With the exception of the island of Lesvos, all the islands where visits were made to farms during our study had been subjected to an eradication program for brucellosis in sheep and goats, whilst such a program had not been applied to the continental area of Greece. It was clear thus, that the risk factors for infection in areas subject to an eradication program were likely to be very different from areas which did not attempt eradication, and for this reason it was decided to omit farmers in the islands (bar in the island of Lesvos) from the analysis of predictors.

Goatherds reported that they had brucellosis more frequently than shepherds. Goats are managed more often in grazing conditions, which leads to coming in contact with animals in other herds, thus increasing the risk of transmission of the pathogen between animals [35] and in turn the risk of transmitting it to farmers. Moreover, *Brucella* spp. are present in the genital-tract excretions of female goats for a longer period post-partum (2–3 months) than in excretions of ewes (for up to 3 weeks) [36]; also, persistent mammary infection with bacterial shedding in milk in subsequent lactation periods is common in goats, whilst in sheep the infection is self-limiting and rarely involves long-standing bacterial excretion [37]. All the above contribute to an increased burden of the pathogen in the environment in goat farms and enhance the risk of human infection.

The application of hand-milking in farms was found to increase the risk of infection of farmers with brucellosis. This is reasonable, as the pathogen is excreted in milk. During hand-milking, farmers touch the teats and milk the animals and *B. melitensis* can be transferred to their hands [36], thus contributing subsequently to their own infection (e.g., consuming food immediately after completion of milking). It is noteworthy that whilst machine-milking has been found to contribute to the quality of milk produced, e.g., bulk-tank milk from machine-milked ewes had lower somatic cell counts [38], the present results also point to the importance of machine-milking for the health of the farmers.

Moreover, the availability of an area to be used as an ‘obstetrical ward’, with animals that stayed there for a short period after parturition, would contribute to creating therein very high burdens of *Brucella*. Therefore, when farmers attend to the newborn lambs/kids (e.g., to support them sucking) or care for the females post-partum (e.g., to evaluate milk production), they could be exposed to a high burden of bacteria and thus have increased chances of infection, even through the respiratory route, a means that can occur in heavily infected environments [39]. Although availability of such an area is important for supporting the survival of newborn lambs/kids and for establishing a dam-newborn bond [40], the present findings nevertheless indicate an increased health risk posed to the farmers and the need to implement increased biosecurity measures in such cases. The findings have a similarity with studies, in which it was found that farmers who performed obstetrical manipulations in their animals (cattle [41]; sheep [15]) were at higher risk of infection with brucellosis. It is noteworthy that the National Public Health Organisation (NPHO) of Greece has reported a seasonality of the infection in people, mostly from April to July [12]. There is a compatibility of those reports with our data, as related specifically to farmers, given that the majority of lambings/kiddings in Greece occur in February to April, i.e., before the Orthodox Easter period. Hence, infection occurs during that season (associated with lambings and kiddings in the farms) and is diagnosed (taking into account the incubation period and the time necessary for testing etc.) 2 to 4 months later. However, for the general human population, it should be noted that the most common route of infection is the foodborne infection.

Cats are not considered to be a major source of infection with *Brucella* spp. for farm animals and people, but they may nevertheless play a role in the persistence of infection within a farm [42]. One may postulate that in dairy sheep farms cats become infected by consuming waste or discarded milk from infected ewes. Cats can be infected and can contribute to the maintenance of the pathogen within a farm, as well as to the spillover of the infection to and from wild animals. It is noteworthy that in a previous study in Greece, evidence of DNA of *B. melitensis* was found in fleas recovered from cats [43]. The association of cats with the occurrence of brucellosis in people may lead to various hypotheses; first, the presence of cats contributes to maintenance of the infection within a farm, thus farmers may become infected more easily during their professional tasks; second, farmers may become infected when cuddling infected cats and, third, farmers may become infected when infested by fleas which often had originated from cats.

It was interesting to find that application of biosecurity-related practices was more frequent among farmers in the continental area of the country than in the islands (as reflected by the mean biosecurity scores in the respective areas). We postulate that as farmers in the latter area felt ‘safer’, due to the control of brucellosis, they were less careful regarding application of biosecurity practices. The significantly higher mean scores in farmers, who reported occurrence of brucellosis, could be related with an appreciation of the importance of biosecurity by these farmers, who had experienced a zoonotic infection in the past. The identification of the isolation of sick animals as the most important component of the biosecurity score can indicate that farmers understand the importance of direct contact with sick animals as a potential means of pathogen transmission.

Reports from around the world indicate the existence of gaps in sheep and goat farmers’ knowledge about biosecurity and application of relevant practices in their farms, e.g., Australia [44]; Finland [45]; Thailand [46] and the United Kingdom [47]. The results of these papers indicate that farmers’ lack of knowledge about biosecurity principles and regulations has not been attended to appropriately.

The present work is a clear illustration of the ‘One-Health’ context and the interactions between animals and human health within this context. ‘One Health’ is the notion that the health of people, animals and ecosystems are strongly interconnected. The definition summarizes the idea that human health and animal health are interdependent and the areas of relevant work include the control of zoonoses [48].

## 5. Conclusions

Brucellosis was reported to be the most frequent zoonotic problem in small ruminant farmers; it was reported significantly more frequently by goat farmers. Management factors applied in the farms have been identified as predictors for the infection in the farmers, for example, machine-milking, the presence of cats in the farms and the presence of a dedicated area for obstetrical cases. Biosecurity scores were higher in farms where farmers reported occurrence of brucellosis in people, so we postulate that these farmers understood better the importance of biosecurity, as they had experienced a zoonotic infection in the past. In farms where the above predictors prevail, farmers should be warned of an increased potential risk for human infection and appropriate biosecurity measures should be implemented.

## Figures and Tables

**Table 1:** Frequency of reported occurrence of zoonotic problems by small ruminant farmers (*n* = 444) during a countrywide study in Greece.

Zoonotic Problem	Sheep Farmers	Goat Farmers
	Who Reported Occurrence of the Problem (*n*)	
Brucellosis	35	22
Contagious ecthyma	2	1
Fungal (*Trichophyton*) infection	1	1
Hydatid disease	1	1
Ophthalmic myiasis	1	1
Anthrax	1	0
Total	41 (12.6%)	26 (21.8%)

**Table 2:** Frequency of reported occurrence of brucellosis by small ruminant farmers (*n* = 444) in different parts of Greece.

Part of the Country (Number of Farmers Interviewed)	Farmers Who Reported Brucellosis
South (*n* = 99)	17 (17.1%)
Central (*n* = 163)	24 (14.7%)
North (*n* = 123)	15 (12.2%)
Islands (*n* = 59)	1 (1.7%)

**Table 3:** Results of multivariable analysis for occurrence of brucellosis in sheep and goat farmers in Greece.

Variables	Odds Ratios ^1^ (95% CI)	*p*
**Sheep Farmers**		
Application of machine- or hand-milking		0.005
Machine-milking (9.0%, 22/244)	reference	-
Hand-milking (22.8%, 13/57)	2.981 (1.397–6.363)	0.005
Availability of cats in the farm		0.007
No (3.7%, 3/82)	reference	
Yes (14.6%, 32/219)	4.506 (1.341–15.147)	0.015
Availability of a separate lambing area		0.009
No (7.5%, 10/133)	reference	-
Yes (14.9%, 25/168)	2.150 (0.994–4.653)	0.05
**Goat Farmers**		
Availability of a separate lambing area		0.001
No (9.3%, 5/54)	reference	-
Yes (32.7%, 17/52)	4.760 (1.605–14.121)	0.005

^1^ Odds ratio calculated against the associations with the lowest prevalence of the variable.

## Data Availability

Most data presented in this study are in the Supplementary Materials. The remaining data are available on request from the corresponding author. Some of the data are not publicly available as they form part of the PhD thesis of the first author, which has not yet been examined, approved and uploaded in the official depository of PhD theses from Greek universities.

## Supplementary Materials

The following supporting information can be downloaded at: https://www.mdpi.com/article/10.3390/ijerph191610372/s1, Table S1: Variables evaluated for potential association with confirmed diagnosis of brucellosis in small ruminant farmers (*n* = 444) during a countrywide study in Greece; Table S2: Details of multivariable models employed for the evaluation of various variables for potential association with reported occurrence of brucellosis in sheep and goat farmers in Greece; Table S3: Biosecurity-related factors applied in 444 small ruminant farms that were evaluated for potential association with reported occurrence of brucellosis during a countrywide study in Greece and scores assigned for each practice aligning or opposing biosecurity rules; Table S4: Results of univariable associations of socio-demographic, farm management and geographic factors with reported occurrence of brucellosis in sheep (*n* = 282) or goat (*n* = 103) farmers during a countrywide study in Greece; Table S5: Estimation of the total number of small ruminant farmers in the country who had had brucellosis at some point during their professional life.
