# Supplementary material for: Zoonotic Problems Reported by Sheep and Goat Farmers and Factors Potentially Contributing to the Occurrence of Brucellosis among Them
Source: Int J Environ Res Public Health. 2022 Aug 20;19(16):10372. doi: 10.3390/ijerph191610372 (PMC9408422; doi:10.3390/ijerph191610372)
Supplement: Supplementary file 1 [file ijerph-19-10372-s001.zip › ijerph-1815285-supplementary.pdf]

Zoonotic problems reported by sheep and goat farmers in Greece and factors potentially contributing to their occurrence

D.T. Lianou, E. Petinaki, C.K. Michael, A. Skoulakis, P.J. Cripps, E.I. Katsarou,
E. Papadopoulos, C. Billinis, A.I. Katsafadou, V.S. Mavrogianni,
M. Caroprese, and G.C. Fthenakis

Table S1. Variables evaluated for potential association with confirmed diagnosis of brucellosis in small ruminant farmers (*n* = 444) during a countrywide study in Greece.

Farmer's gender (male / female)
Length of farming activity (years)
Farmer's professional involvement in farming (full-time / part-time)
Farmer's daily time spent at the farm (hours)
Farmer's education (primary / secondary / post-secondary vocational / tertiary)
Farmer's family farming tradition (yes / no)
Management system applied in the farm (description according to EFSA classification)[1]
Transhumance (yes / no)
Application of machine- or hand-milking
Number of animals in the farm (no.)
Presence of large ruminants (cattle, buffaloes) in the farm (yes / no)
Presence of pigs in the farm (yes / no)
Presence of dogs in the farm (yes / no)
Presence of cats in the farm (yes / no)
Presence of equines (horses, donkeys, mules, hinnies) in the farm (yes / no)
Breed of animals in the farm (crossbreeds / imported / local)
Annual veterinary visits to the farm (no).
Daily number of milking sessions applied in the farm (no.)
Annual occasions of disinfections performed in the farm (no.)
Application of reproductive control practices in the farm (yes / no)[2]

Availability of a separate lambing / kidding area
Vaccination against brucellosis applied in the farm (yes / no)
Distance of farm from hospital or clinic (km)

1. Classification according to the European Food Safety Authority system (European Food Safety Authority. Scientific opinion on the welfare risks related to the farming of sheep for wool, meat and milk production. *EFSA J.* **2014**, *12*, 3933-4060).
2. In this context, reproductive control practices refer only to intravaginal application of progesterone sponges or devices.

Table S2. Details of multivariable models employed for the evaluation of various variables for potential association with reported occurrence of brucellosis in sheep and goat farmers in Greece.

Outcome	Variables offered to the multivariable models (n)	Variables required in the final models
Occurrence of brucellosis - sheep farmers	8	(a) Application of machine- or hand-milking, (b) Presence of dogs in the farm, (c) Presence of cats in the farm, (d) Annual veterinary visits to the farm, (e) Availability of a separate lambing area
Occurrence of brucellosis – goat farmers	7	(a) Farmer's education, (b) Management system applied in the farm, (c) Annual veterinary visits to the farm, (d) Availability of a separate kidding area

Table S3. Biosecurity-related factors applied in 444 small ruminant farms that were evaluated for potential association with reported occurrence of brucellosis during a countrywide study in Greece and scores assigned for each practice aligning or opposing biosecurity rules.

Biosecurity-related practice	Score assignment according to the practices applied	
	**Score '1'**	**Score '0'**
Quarantine of new animals arriving at the farm	Yes	No
Isolation of sick animals at the farm	Yes	No
Means for disposal of carcasses of animals that died in the farm	Incineration / Burial / Removal by specialised agent	Given to dogs / Left unburied / Left in water streams
Presence of a ditch at the entrance of the farm	Yes	No
Presence of a fence or a wall around the farm	Yes	No
Carrying out disinfections in the farm	Yes	No

Table S4. Results of univariable associations of socio-demographic, farm management and geographic factors with reported occurrence of brucellosis in sheep (n=282) or goat (n=103) farmers during a countrywide study in Greece.

**A. Sheep farmers (*n* = 301)**								
**Farmers with no occurrence of brucellosis (*n* = 266)**				**Farmers with occurrence of brucellosis (*n* = 35)**				***P***
Farmer's gender								
Female		Male		Female		Male		
5.6% (15/266)		94.4% (251/266)		5.7% (2/35)		94.3% (33/35)		0.99
Farmer's age								
46.8±1.2 years				49.7±2.2 years				0.19
Length of farming activity								
23.0±1.8 years				29.2±2.5 years				0.036
Farmer's professional involvement in farming								
Full-time		Part-time		Full-time		Part-time		
86.0% (229/266)		14.0% (27/266)		94.3% (33/35)		5.7% (2/35)		0.37
Farmer's daily time spent at the farm								
11.4±0.6 hours				12.6±0.7 hours				0.11
Farmer's education								
Primary	Secondary	Vocational	Tertiary	Primary	Secondary	Vocational	Tertiary	
24.4% (65/266)	44.7% (119/266)	18.0% (48/266)	12.8% (34/266)	34.3% (12/35)	40.0% (14/35)	11.4% (4/35)	14.3% (5/35)	0.54

Farmer’s family farming tradition								
Yes		No		Yes		No		
86.5% (230/266)		13.5% (36/266)		91.4% (32/35)		8.6% (3/35)		0.41
Management system applied in farms								
Intensive	Semi-intensive	Semi-extensive	Extensive	Intensive	Semi-intensive	Semi-extensive	Extensive	
15.0% (40/266)	46.7% (124/266)	34.6% (92/266)	3.7% (10/266)	8.6% (3/35)	45.7% (16/35)	40.0% (14/35)	5.7% (2/35)	0.68
Transhumance								
Yes		No		Yes		No		
13.2% (35/266)		86.8% (231/266)		11.4% (4/35)		88.6% (31/35)		0.77
Application of machine- or hand-milking								
Machine-milking		Hand-milking		Machine-milking		Hand-milking		
83.5% (222/266)		16.5% (44/266)		61.8% (22/35)		38.2% (13/35)		0.003
Number of animals in the farm								
325±18 animals				312±28 animals				0.77
Breed of animals in the farm								
Crossbreeds	Imported	Local		Crossbreeds	Imported	Local		
25.6% (68/266)	38.0% (101/266)	36.5% (97/266)		31.4% (11/35)	22.9% (8/35)	45.8% (16/35)		0.22
Annual veterinary visits to the farm								
7.7±0.6 visits				5.7±0.8 visits				0.11

Daily number of milking sessions applied in the farm				
2.2±0.0 milking sessions		2.3±0.1 milking sessions		0.33
Annual occasions of disinfections performed in the farm				
3.5±0.2 occasions		3.0±0.4 occasions		0.43
Application of reproductive control practices in the farm				
Yes	No	Yes	No	
31.2% (83/266)	68.8% (183/266)	28.6% (10/35)	71.4% (25/35)	0.75
Availability of a separate lambing area				
Yes	No	Yes	No	
53.8% (143/266)	46.2% (123/266)	71.4% (25/35)	28.6% (10/35)	0.048
Vaccination against brucellosis applied in the farm				
Yes	No	Yes	No	
100% (266/266)	0.0% (0/266)	100% (35/35)	0.0% (0/35)	n/a
Distance from hospital or clinic				
14.7±0.4 km		13.7±1.2 km		0.44
Presence of large ruminants in the farm				
Yes	No	Yes	No	
9.8% (26/266)	90.2% (240/266)	11.4% (4/35)	88.6% (31/35)	0.76

Presence of pigs in the farm				
Yes	No	Yes	No	
12.8% (34/266)	87.2% (232/266)	5.7% (2/35)	84.3% (33/35)	0.23
Presence of dogs in the farm				
Yes	No	Yes	No	
92.5% (246/266)	7.5% (20/266)	100% (35/35)	0.0% (0/35)	0.09
Presence of cats in the farm				
Yes	No	Yes	No	
70.3% (187/266)	29.7% (79/266)	91.4% (32/35)	8.6% (3/35)	0.008
Presence of equines in the farm				
Yes	No	Yes	No	
18.0% (48/266)	82.0% (218/266)	20.0% (7/35)	80.0% (28/35)	0.78
**B. Goat farmers (*n* = 106)**				
**Farmers with no occurrence of brucellosis (*n* = 84)**		**Farmers with occurrence of brucellosis (*n* = 22)**		***P***
Farmer's gender				
Female	Male	Female	Male	
10.7% (9/84)	89.3% (75/84)	0.0% (0/22)	100.0% (22/22)	0.11
Farmer's age				
46.5±1.4 years		46.8±2.7 years		0.93

Length of farming activity								
23.9±1.8 years				24.8±3.1 years				0.82
Farmer’s professional involvement in farming								
Full-time		Part-time		Full-time		Part-time		
88.1% (74/84)		11.9% (10/84)		81.8% (18/22)		18.2% (4/22)		0.44
Farmer’s daily time spent at the farm								
12.3±0.4 hours				12.6±0.7 hours				0.56
Farmer’s education								
Primary	Secondary	Vocational	Tertiary	Primary	Secondary	Vocational	Tertiary	
23.8% (20/84)	46.4% (39/84)	11.9% (10/84)	17.9% (15/84)	50.0% (11/22)	31.8% (7/22)	9.1% (2/22)	9.1% (2/22)	0.12
Farmer’s family farming tradition								
Yes		No		Yes		No		
84.5% (71/84)		15.5% (13/84)		90.9% (20/22)		9.1% (2/22)		0.44
Management system applied in farms								
Intensive	Semi-intensive	Semi-extensive	Extensive	Intensive	Semi-intensive	Semi-extensive	Extensive	
9.5% (8/84)	29.8% (25/84)	48.8% (41/84)	11.9% (10/84)	0.0% (0/22)	18.2% (4/22)	59.1% (13/22)	22.7% (5/22)	0.19
Transhumance								
Yes		No		Yes		No		
19.0% (16/84)		81.0% (68/84)		40.9% (9/22)		59.1% (13/22)		0.032

<table>
<tr><td colspan="12">Application of machine- or hand-milking</td><td></td></tr>
<tr><td colspan="3">Machine-milking</td><td colspan="3">Hand-milking</td><td colspan="3">Machine-milking</td><td colspan="3">Hand-milking</td><td></td></tr>
<tr><td colspan="3">56.0% (47/84)</td><td colspan="3">44.0% (37/84)</td><td colspan="3">45.5% (10/22)</td><td colspan="3">54.5% (12/22)</td><td>0.38</td></tr>
<tr><td colspan="12">Number of animals in the farm</td><td></td></tr>
<tr><td colspan="6">223±22 animals</td><td colspan="6">250±38 animals</td><td>0.62</td></tr>
<tr><td colspan="12">Breed of animals in the farm</td><td></td></tr>
<tr><td colspan="2">Crossbreeds</td><td colspan="2">Imported</td><td colspan="2">Local</td><td colspan="2">Crossbreeds</td><td colspan="2">Imported</td><td colspan="2">Local</td><td></td></tr>
<tr><td colspan="2">21.4% (18/84)</td><td colspan="2">38.1% (32/84)</td><td colspan="2">40.5% (34/84)</td><td colspan="2">27.3% (6/22)</td><td colspan="2">31.8% (7/22)</td><td colspan="2">40.9% (9/22)</td><td>0.80</td></tr>
<tr><td colspan="12">Annual veterinary visits to the farm</td><td></td></tr>
<tr><td colspan="6">8.1±0.8 visits</td><td colspan="6">6.0±0.9 visits</td><td>0.19</td></tr>
<tr><td colspan="12">Daily number of milking sessions applied in the farm</td><td></td></tr>
<tr><td colspan="6">2.1±0.0 milking sessions</td><td colspan="6">2.0±0.0 milking sessions</td><td>0.39</td></tr>
<tr><td colspan="12">Annual occasions of disinfections performed in the farm</td><td></td></tr>
<tr><td colspan="6">4.0±0.4 occasions</td><td colspan="6">3.4±1.1 occasions</td><td>0.60</td></tr>
<tr><td colspan="12">Application of reproductive control practices in the farm</td><td></td></tr>
<tr><td colspan="3">Yes</td><td colspan="3">No</td><td colspan="3">Yes</td><td colspan="3">No</td><td></td></tr>
<tr><td colspan="3">16.7% (14/84)</td><td colspan="3">83.3% (70/84)</td><td colspan="3">4.5% (1/22)</td><td colspan="3">95.5% (21/22)</td><td>0.15</td></tr>
<tr><td colspan="12">Availability of a separate kidding area</td><td></td></tr>
<tr><td colspan="3">Yes</td><td colspan="3">No</td><td colspan="3">Yes</td><td colspan="3">No</td><td></td></tr>
<tr><td colspan="3">41.7% (35/84)</td><td colspan="3">58.3% (49/84)</td><td colspan="3">77.3% (17/22)</td><td colspan="3">22.7% (5/22)</td><td>0.003</td></tr>
</table>

Vaccination against brucellosis applied in the farm				
Yes	No	Yes	No	
100% (84/84)	0.0% (0/81)	100.0% (22/22)	0.0% (0/22)	n/a
Distance from hospital or clinic				
17.3±1.2 km		16.6±1.6 km		0.76
Presence of large ruminants in the farm				
Yes	No	Yes	No	
11	73	2	20	0.61
Presence of pigs in the farm				
Yes	No	Yes	No	
12	72	2	20	0.52
Presence of dogs in the farm				
Yes	No	Yes	No	
81	3	22	0	0.37
Presence of cats in the farm				
Yes	No	Yes	No	
60	24	16	6	0.90
Presence of equines in the farm				
Yes	No	Yes	No	
18	66	6	16	0.56

Table S5. Estimation of the total number of small ruminant farmers in the country who had had brucellosis at some point during their professional life.

**Proportion (95% confidence intervals) of farmers in the studied sample, who had had brucellosis during their professional life**		
Area of the country	Sheep farmers	Goat farmers
Central	17/127<br>0.134 (0.085 – 0.204)	7/36<br>0.194 (0.098 – 0.350)
Islands	1/43<br>0.023 (0.004 – 0.121)	0/16<br>0.000 (0.000 – 0.194)
North	10/87<br>0.115 (0.064 – 0.199)	5/36<br>0.139 (0.061 – 0.287)
South	7/68<br>0.103 (0.051 – 0.198)	10/31<br>0.323 (0.186 – 0.499)
**Number of small ruminant farms available in the country in 2019**		
Area of the country	Sheep farms	Goat farms
Central	13037	3660
Islands	8970	1837
North	13420	4763
South	6860	2771
**Total number (95% confidence intervals) of small ruminant farmers in the country, who had had brucellosis during their professional life**		
Area of the country	Sheep farmers	Goat farmers
Central	1746 (1112 – 2660)	712 (357 – 1283)
Islands	209 (37 – 1083)	0 (0 – 356)
North	1542 (854 – 2668)	662 (290 – 1365)
South	706 (348 – 1355)	894 (515 – 1382)
	4203 (2351 – 7766)	2268 (1162 – 4386)
